# Observation on Pain Release by Long-Round Needle Therapy in Knee Osteoarthritis Related with Meridian-Sinews Theory

**DOI:** 10.1155/2013/781289

**Published:** 2013-08-20

**Authors:** Yuan-shi Liu, Li-gong Xue, Xiao-jian Ma, Chun-shan Liu

**Affiliations:** ^1^Guang'anmen Hospital, China Academy of Chinese Medical Sciences, Beijing 100053, China; ^2^Institute of Acupuncture and Moxibustion, China Academy of Chinese Medical Sciences, Beijing 100700, China; ^3^Shandong Laizhou Maternity and Child Care Hospital, Laizhou 261400, China

## Abstract

To evaluate the effectiveness of long-round needle therapy for pain relief in patients with knee osteoarthritis, 192 patients were included in a multicenter, randomized, controlled trial. 97 patients were randomized to the long-round needle therapy group (EG), and 95 patients were randomized to the control group (CG). In EG, the long-round needle therapy was performed once every 7 days for 3 therapy sessions. Ibuprofen sustained-release capsules were administered orally in CG, 1 pill each time, twice daily for 3 weeks. Curative effect was measured after the therapy and was evaluated at a 3-month follow-up interview. In EG, the treatment resulted in a basic cure for 79 patients, was effective for 15 patients, and was ineffective for 1 patient. In CG, the treatment resulted in a basic cure for 30 patients, was effective for 38 patients, and was ineffective for 21 patients. In the follow-up examination in EG, 75 patients were basically cured, and the treatment was effective for 11 patients and ineffective for 9. In CG, 22 were basically cured, 31 found the treatment effective, and 36 found the treatment ineffective. The curative effects in EG after both the treatment and the 3-mouth followup were significantly more superior than that in CG (*P* < 0.01) which should be adopted more widely.

## 1. Introduction

Knee osteoarthritis (KOA) is prevalent in the elderly population [1]. It is characterized clinically by pain and stiffness, especially when walking up and down stairs, and has a severe, negative impact on quality of life. Pathologically, osteoarthritis is characterized by degeneration of articular cartilage, bone hypertrophy, and subchondral sclerosis [2]. The aim of treatment of KOA is mainly to control symptoms and includes the use of nonsteroidal anti-inflammatory drugs, glucosamine, topical analgesics, intra-articular injection of sodium hyaluronate, and surgical treatment [3]. Many patients find the results of these therapies less than satisfactory; however, more effective treatments have not been developed to date. Since ibuprofen sustained-release capsules (Fenbid) appeared on the market in the 1970s, it has become the drug of choice of KOA [4]. 

Long-round needle therapy has its roots in the Han Dynasty classic of Chinese medical theory, the Yellow Emperor's Inner Canon, specifically in the “Nine Needles” chapter [5]. Long-round needle therapy was developed by Xue in 1998 and has since become a popular therapy in China [6, 7]. It is indicated primarily for various types of chronic pain, such as secondary prolapse of lumbar intervertebral disks [8] and shoulder muscle strain [9]. The purpose of this research is to compare the effectiveness of long-round needle therapy and conventional Fenbid for knee osteoarthritis using a multicenter, randomized, controlled trial (RCT) design. Secondarily, we hope that this research will contribute to a hypothesis on the mechanism of action of long-round needle therapy based on traditional meridian-sinews theory.

## 2. Objects and Methods

### 2.1. Subjects

192 patients with KOA were recruited from (1) the out-patient clinic or in-patient department of Institute of Acupuncture and Moxibustion, China Academy of Chinese Medical Sciences; (2) Guang'anmen Hospital, China Academy of Chinese Medical Sciences, or (3) Shandong Laizhou Maternity and Child Care Hospital. For inclusion, all patients met the following criteria: (1) met the diagnostic criteria of KOA [10, 11], (2) aged 40–65, and (3) signed informed consent. Exclusion was based on the following criteria: (1) did not meet the diagnostic criteria of KOA, (2) serious cardiocerebrovascular system diseases, and (3) unwilling to accept the treatment. Suspension criteria included (1) inability to complete the treatment, (2) not complying with treatment protocols, and (3) serious complications.

### 2.2. Data Collection and Test Design

192 patients were included in the trial. 97 patients were randomized to the long-needle group (EG), and 85 were randomized to the pharmacotherapy control group (CG). The trial was conducted according to Good Clinical Practice guidelines, and the rights of patients were protected at all times. The study design was a multicenter, randomized, controlled trial. The sample size was calculated based on the effective rate derived from a review of the literature (*α* = 0.05, *β* = 0.01). We determined that sample size should be *n* = 192, assuming a withdraw rate of 15%, and with a target of 180 patients. Random numbers were acquired from Statistics Analysis System, EG-CG with a 1 : 1 ratio. The random number table was based on the order in which patients joined the study. The concealment method for the random allocation scheme was the total allocation scheme concealment method, including allocating randomization numbers in order and placing them in opaque envelopes. All 192 patients were randomized to either group EG or CG. The trial began after receiving Ethics Committee approval, and informed consent was obtained from all patients.

### 2.3. Intervention and Methods

#### 2.3.1. EG

The needle instrument used was the long-round needle. Sites along the pathways of meridian-sinews were selected for treatment based on traditional diagnostic methods for meridian-sinew disorders. The sites were primarily sites determined to be “knotted,” and needle manipulation was aimed at releasing these knots. 

*Site Selection. *Sites along the meridian-sinew of Foot Tai-yang: sub-BL39 (lateral end of the fossa popliteal crease, on the medial border of biceps muscle of the thigh), sub-BL40 (in the middle of the fossa popliteal crease), and sub-BL55 (on the posterior leg, lower point of middle of lower fossa popliteal, and at the level of the inferior border of caput fibulae).

Sites along the meridian-sinew of Foot Shao-yang: intertibiofibulae (on the lateral knee, at the joint space of knee), caput fibulae (superior border of caput fibulae), and subtibia (lateral femur, at lateral condyle of femur).

Sites along the meridian-sinew of Foot Yang-ming: lower outer patella (lower outer border of patella), external condyle of tibia (external tuberosity of tibia), lower inner patella (lower inner border of patella, origin of patella medial accessory ligament), inner malleolus condyle of tibia (medial tuberosity of internal epicondyle tibia), and lower patella (lower border of the patella).

Sites along the three Yin meridian-sinews of the foot: upper SP9 (on the medial surface of the leg, at medial surface of the medial condyle of the tibia, and at the level of tubercle of tibia), sub-LR7 (on the medial surface of the leg, at medial border of the medial condyle of the tibia), intercoronary patella (on the lateral knee, in the joint space of the knee), subcoronary crevice (medial surface of the leg, medial surface of the medial condyle of femur), and sub-LR8 (medial knee, superior to the medial condyle of the tibia, and at the bend of gracilis tackle).

*Technique.* Needling techniques for “knot release”:

1. Joint puncture: insert needle until the surface of the sinew-knot lesion is reached, then scrape and pluck, moving right to left in order to release superficial adhesions.
2. Soothing puncture: insert the needle deeply into the center of adhesions on the sinew-knot lesions located next to tendons. Then slant the tip of the needle upwards, scraping and lifting the adhesions all around it in order to release pressure.

The needling technique employed was chosen based on the location of the meridian-sinew site. Pressure was applied to all sites after needling in order to stop bleeding. To prevent infection, a sterile dressing was applied and left in place for two days.

The therapy above was performed once every 7 days for a total of 3 sessions. Curative effect was measured after the 3 sessions were completed, and long-term results were evaluated with a followup 3 months later.

#### 2.3.2. CG

Ibuprofen sustained-release capsules (Fenbid) (0.3 g ibuprofen per pill) were administered orally to patients in CG, 1 pill per dose, twice daily for 3 weeks. Curative effect was measured after 3 weeks, and long-term results were evaluated with a followup 3 months later.

### 2.4. KOA Diagnosis and Evaluation of Treatment Results

KOA diagnosis was based on the *Clinical and Radiological Reference from the American College of Rheumatology* [10]. Guidelines for measuring therapeutic effectiveness were based on the official People's Republic of China,* Standards for Diagnosis and Therapeutic Effectiveness TCM Patterns and Diseases*, specifically the TCM orthopedics standards for treatment effectiveness for knee osteoarthritis (ZY/T 001.8-94) [11]. Taking into account that the aforementioned standard is not sufficiently quantitative, we introduced therapeutic effectiveness grading and grading index. We developed a KOA treatment observation table to record pain, discomfort, morning stiffness, and walking distance. Discomfort was graded as absent, slight, moderate, or severe and based on the Lequesne evaluation of KOA severity and mobility index. Every item was recorded before treatment. We used the following formula (nimodipine formula): *n* (Therapeutic Effectiveness index) = [(Grade before treatment − Grade after treatment) ÷ Grade before treatment] × 100%. Recover: *n*⩾90%; effective: *n* is 30%–90%; ineffective: *n* < 30%.

### 2.5. Statistical Analysis

All analysis was completed by the National Clinical Experimental Center for New Chinese Herbal Drugs (Beijing, GCP center) by researchers who had not taken part in the clinical trial design or research. First, RCDMS was used to enter and lock data. Second, data was transferred to SPSS 11.5 after confirmation. Then, the analysis, including pain grading calculation and therapeutic effectiveness, was performed, and the clinical data was examined separately. *P* < 0.05 was used as the standard for statistical significance.

## 3. Results

### 3.1. Comparison of Basic Data between the Two Groups

A total of 8 patients were removed from the study for noncompliance. Two patients in the EG were removed because they began the use of medication during the study period, and 6 patients in the CG were removed for taking other pain killers during the study period. There were no suspended cases or off cases in both groups. The rate of loss was less than 20%. The number of patients was adequate for the trial (Table 1). There was no significant differences in sex, age, or duration of illness (*P* > 0.05) between the two groups; therefore, the groups were comparable.

### 3.2. Therapeutic Evaluation

Therapeutic effectiveness at the end of treatment is shown in Table 2. Therapeutic effectiveness 3 months after the completion of treatment is shown in Table 3.

As seen in Table 2, *t* = −9.038, *P* < 0.01; therefore, the difference is significant, and the therapeutic effectiveness of EG is superior to the CG.

As is seen in Table 3, *t* = −10.219, *P* < 0.01; therefore, the difference is significant, and the therapeutic effectiveness of EG is superior to the CG.

## 4. Discussion

The results showed that long-round needle therapy was superior to Fenbid after both a 3-week course of treatment and at 3-month followup. 

Osteoarthritis of the knee, known as impediment (*bi*) disease in TCM, is common in the elderly. It is characterized by joint pain and difficulty of movement and may also lead to economic loss and interfere with activities of daily living (ADL). In modern biomedicine, diagnosis is based on X-ray examination confirming cartilage injury (articular cartilage injury, hyperosteogeny, etc.). The main treatments are prosthetic replacement, cartilage prosthesis, and spur removal. Fenbid is the internationally recognized pain killer most often used in nonoperative treatment. Although it is reasonably effective at relieving pain, many patients find the side effects unacceptable. Therefore, other therapies are urgently needed.

In the authors' opinion, because there is no nociceptive nerve endings in articular cartilage, KOA pain is not caused by articular cartilage injury but is due to meridian-sinew injury. Long-term, repetitive strain causes “knots” in the meridian-sinews, which in turn block the flow of Qi-blood. It is the knot which causes arthralgia at the joint in the elderly. Therefore, it is necessary to choose treatment sites based on traditional syndrome differentiation and to release the knots. If this is done effectively, it can stop pain and help patients avoid surgery [12].

Based on the meridian-sinews theory in the meridian-sinew chapter of the Yellow Emperor's Canon [13], there are 6 meridian-sinews around the knee. They are meridian-sinew of Foot Tai-yang, meridian-sinew of Foot Shao-yang, meridian-sinew of Foot Yang-ming, meridian-sinew of Foot Tai-yin, meridian-sinew of Foot Jue-yin, and meridian-sinew of Foot Shao-yin. The *Spiritual Pivot* section of the Yellow Emperor's Canon contains a detailed description of the distribution and collection of every meridian-sinew around the knee. These distribution and collection sites correspond to the most common sites of KOA pain.

There is a close relationship between meridian-channels and meridian-sinews. Meridian-channels are the sites of Qi-blood flow, and these channels also exist within meridian-sinews. Any disorder of the meridian-sinews, such as knots or strips, will obstruct the flow of Qi and cause pain. The author applied the long-round needle, which is an innovation based on the description of the long needle and the round needle in the Yellow Emperor's Canon of Internal Medicine [4], and the “knot release” method in the same book. Knot release can reduce fluid pressure and therefore reduce or even eliminate the collection and seepage of fluid and humors. Treating the meridian-sinews can enhance the flow of interstitial fluid along the meridian-channels, thereby improving overall functioning [14], increasing the elimination of toxins, and reducing pain. The comparison in this paper showed that long-round needle therapy was superior to Fenbid for treating the pain of KOA and should therefore be more widely adopted as a treatment.

## Figures and Tables

**Table 1:** Data summary for patient numbers in the two groups (EG and CG).

Group	In	Suspended	Removed	Off	Qualified	Rate of qualified (%)
EG	97	0	2	0	95	97.94
CG	95	0	6	0	89	93.68

**Table 2:** Therapeutic effectiveness at end of treatment.

Group	*N*	Mean	Standard deviation	Median	Minimum	Maximum	*t*	*P*
EG	95	47.462	8.116	3.584	1	14	−9.038	0.000
CG	89	38.901	3.214	3.773	0	14		

**Table 3:** Therapeutic effectiveness at followup 3 months after treatment completion.

Group	*N*	Mean	Standard deviation	Median	Minimum	Maximum	*t*	*P*
EG	95	47.462	9.3158	2.34867	−4	14	−10.22	0.000
CG	89	38.901	4.8427	3.50940	−4	12		
